# Schizoaffective Disorder and Concurrent Rhabdomyolysis

**DOI:** 10.7759/cureus.19896

**Published:** 2021-11-25

**Authors:** Thomas Varkey, Christopher Demetriades, Natalie Malluru, Zachary I Merhavy, Kyle Simtion, Caitlyn Garmer, Colton Zeitler, Raaj Pyada, Anne M Nguyen, Jack B Ding

**Affiliations:** 1 Colangelo College of Business, Grand Canyon University, Phoenix, USA; 2 Department of Neurology, Dell Seton Medical Center, Austin, USA; 3 Department of Psychology, Dell Medical School, The University of Texas at Austin, Austin, USA; 4 Department of Internal Medicine, Dell Medical School, The University of Texas at Austin, Austin, USA; 5 Department of Internal Medicine, Ross University School of Medicine, Bridgetown, BRB; 6 Department of Internal Medicine, Lincoln Memorial University, DeBusk College of Osteopathic Medicine, Harrogate, USA; 7 Department of Internal Medicine, Arizona College of Osteopathic Medicine, Midwestern University, Glendale, USA; 8 Department of Internal Medicine, The University of Adelaide, Adelaide, AUS; 9 Department of Internal Medicine, Royal Adelaide Hospital, Adelaide, AUS

**Keywords:** concurrent disorders, law enforcement, interprofessional care, schizoaffective disorder, non-traumatic rhabdomyolysis

## Abstract

The authors present a unique case of schizoaffective disorder exacerbation, complicated by substance misuse, rhabdomyolysis, and acute renal injury. The patient had been recently released from jail and was not on any psychiatric medications aside. His family reported bizarre behavior involving the patient spending a significant amount of time in an outdoor hot tub exposed to extreme heat, which the patient justified as necessary to protect him from snakes. The patient was diagnosed with severe dehydration and rhabdomyolysis, both of which were managed by the primary care team in a hospital setting with specialist input from the psychiatry and renal departments. The patient exhibited paranoid ideations toward the medical team and at times was agitated and combative. Resolution of this distrust was pivotal to successful treatment and was made possible through trilateral communication between the patient, the police officers, and medical staff.

## Introduction

Schizoaffective disorder affects many patients; however, epidemiological data have been hard for the scientific community to gather as the definition and diagnostic criteria have been changed multiple times since its initial inclusion within the Diagnostic and Statistical Manual of Mental Disorders [1]. Because of the underlying psychiatric disorder, many patients often suffer from a lack of treatment or have difficulty during episodes receiving adequate care for their co-morbid conditions [1]. Herein, the authors provide a clinical case study and discuss management techniques that were utilized to provide the best care for the patient who suffered from concurrent schizoaffective disorder and rhabdomyolysis.

## Case presentation

A 35-year-old male presented with chief complaints of severe back pain and dehydration. Hours earlier, his family found him floating in a hot tub outside his parent’s suburban home, exposed to the midday summer heat of central Texas. His past medical history was pertinent for bipolar type schizoaffective disorder and substance use disorder (methamphetamines, marijuana, and cocaine). In the past, he had been admitted for his schizoaffective disorder and recently had been released from a correctional facility where he had been incarcerated for drug-related offenses. Collateral history from the patient’s family revealed the patient was recently released from a correctional facility and missed an appointment to receive a long-acting injectable antipsychotic (risperidone), but he was otherwise unmedicated and had no psychiatry follow-up. He was brought to the emergency department under a police order of emergency detention (POED) with chief complaints of extreme back pain and the sense that his house was “full of snakes.” The patient requested the medical team to perform a “full-body transplant” to alleviate his back pain and so he would be able to move on to “a new life.” The patient disclosed that he had been in the hot tub for several hours because it would, “protect [him] from the mind-controlling electric machine which creates these snakes that attack [him].” The patient denied any recent recreational or illicit substance use. The remainder of the history was deferred due to the patient’s psychosis. Initially, the patient was infused with two liters of Ringer's lactate solution in the emergency room and given a sitter to prevent him from leaving the patient care room.

Physical examination

On general inspection, the patient had a first-degree burn covering his bilateral upper torso, arms, and calves. He smelled of salt and appeared very dehydrated with dry mucus membranes of the mouth. Capillary refill was delayed to roughly four seconds and his forehead was covered in a fine coat of salt. Vital signs were pertinent for sinus tachycardia to the 120s and systolic blood pressure in the 110s/60s (mmHg). However, the patient was hemodynamically stable and did not require intensive stabilization or surgical intervention.

Laboratory results

A complete blood count (CBC), complete metabolic panel (CMP), urinalysis, toxicology screen, and HIV test were obtained. The patient’s urine sample was a dark brown color, like that of a dark tea, which led the team to order a creatine kinase (CK) level. The patient’s CK serum level was elevated at approximately 90,000 mg/dL (22 to 198 mg/dL). The patient’s urinary toxicology screen was positive for both benzodiazepines and methamphetamines. The CMP revealed a creatinine of approximately 5 mg/dL, significantly elevated blood urea nitrogen (BUN), and mild transaminitis, while other laboratory values were within normal limits (Table 1). While awaiting laboratory findings, the primary care team ordered an abdominal ultrasound (US), which demonstrated no acute changes or morphologic alterations to the kidney parenchyma, ureters, or other urinary structures. Furthermore, the abdominal US did not demonstrate findings on the liver, gall bladder, or other gastrointestinal structures. These laboratory values and the imaging study led the primary medicine team to consult the nephrology team for potential hemodialysis for acute renal failure (ARF) secondary to rhabdomyolysis.

**Table 1:** Initial laboratory and imaging findings.

Study	Sub-study	Findings	Normal ranges
Urinalysis	Gross findings	Dark brown color	Light yellow coloration
	Urine dipstick	+Hemoglobin	Negative findings
	Urine toxin screen	Negative	Negative
	Microscopy	No RBCs, trace epithelial cells, or trace WBCs	Negative findings
Basic metabolic panel	Creatinine	5 mg/dL	0.74–1.35 mg/dL
	Blood urea nitrogen	88 mg/dL	6–24 mg/dL
Liver function tests	Aspartate transaminase	903 units/L	14–59 units/L
	Alanine transaminase	213 units/L	7–55 units/L
	Total bilirubin	3.8 mg/dL	0–1.2 mg/dL
	Direct bilirubin	0.7 mg/dL	0–0.3 mg/dL
Creatine kinase		90,679 mg/dL	22–198 mg/dL
Abdominal ultrasound		No acute changes or morphologic alterations to the kidney parenchyma, ureters, or other urinary structures. No findings on the liver, gall bladder, or other gastrointestinal structures	Normal anatomy

Consultant team’s recommendations for management

Based on the patient’s clinical condition, the nephrology team recommended aggressive IV fluid resuscitation at 200 mL/hr to facilitate the renal clearance of myoglobin. The nephrology team acknowledged that if the patient’s creatinine kinase levels continued to increase, they may need to begin hemodialysis. However, they were hopeful that conservative management would permit physiological recovery. As the patient faced a life-threatening condition and clearly lacked the capacity to refuse treatment, the psychiatry team acknowledged that they would likely need to file for an order of protective custody (OPC) to ensure that he would not be able to leave in his altered state, they also stated that they would start 1 mg of oral risperidone given once daily to treat his psychosis and baseline schizoaffective disorder and adjust the medications as needed from there.

Psychiatric complications

Shortly after starting treatment for the ARF, the patient complained that he had no issues with his kidneys and that he wanted a transfer to another location to obtain a new body, exclaiming that “if you don’t even have a muscle regeneration machine, how can you call this a hospital?”. He stated that he did not want the IV catheter placed, the fluids given, or an X-ray taken as he “doesn’t want his plasma taken out.” After his mother arrived, the patient successfully removed his IV catheter and attempted to leave against medical advice (AMA), which he was unable to do secondary to his POED. The patient became increasingly agitated and required 5 mg of haloperidol, 2 mg of lorazepam, and 50 mg of diphenhydramine by intramuscular injection. Despite this, he continued to be agitated and aggressive, which necessitated another intramuscular injection of 5 mg of haloperidol and 2 mg of lorazepam. Reestablishing IV access proved to be very difficult leading to a six-hour delay in resuming fluid infusion.

Over the following days, the patient was amenable to taking oral risperidone. However, he also called the police a total of four times stating that he was being held against his will and that the hospital was working to harm him. These accusations necessitated the medical team to collaborate with the police. With the support of the head of security, the team summoned a police officer from another floor to “answer the call” from the patient. The officer kindly explained that they had received his call, but that he had a legal order to remain in the hospital, and that it was important to listen to the doctors and take his medications. The patient agreed to take an oral dose of risperidone and expressed his willingness to restart the IV fluids. This helped resolve some of the patient’s guardedness, allowing for continued IV restoration of fluid status without the patient removing his catheter. Over the next several days, the patient’s urine output normalized, and laboratory tests returned reassuring serum creatinine levels. The primary care team transitioned the patient from IV fluid replacement to aggressive oral fluid replacement and the IV catheter was removed, which surprisingly alleviated nearly all of the patient’s paranoid ideations and distrust toward the medical team. After a total of 11 days of hospitalization, during which pharmacy, physical therapy, occupational therapy, and case management all worked to ensure his proper physical and social care, the nephrology team and internal medicine team cleared him for discharge to inpatient psychiatry for treatment of his schizoaffective disorder. He was discharged on a 0.1 mg clonidine extended-release patch to be replaced weekly, 1 mg of risperidone taken orally twice daily, and 800 mg of sevelamer to be taken three times a day with meals. He and his parents were given education regarding his adherence to his medications, major signs and symptoms associated with medication use, and finally his rehabilitation plan for after the psychiatric hospitalization was discussed with the patient and his case manager.

## Discussion

The main clinical pearl here is early recognition of problems and immediate action by the clinical care team. Within minutes of the patient’s admission, both the nephrology and the psychiatry team were consulted for the management of the patient’s co-morbid conditions. Additionally, early interdisciplinary care by pharmacy, physical therapy, occupational therapy, and case management ensured that the patient’s medical and psychosocial care was optimized. Due to the interdisciplinary care and quick notification of the patient’s needs, the different teams effectively worked to prevent permanent damage to his renal functionality. Lastly, the team ensured he was discharged to be treated for the psychiatric illness, which had led to his initial presentation, the schizoaffective disorder. This early consultation and practice by interdisciplinary teams lead to a lower readmission rate, higher patient satisfaction, and decreased mortality and morbidity in patients [2-5].

The second clinical pearl is the realization that non-medical personnel can act as members of the care team. While the use of interdisciplinary teams and the appreciation of the hard work that they do has become more universal [6-9], in the patient’s care, there was a need for peacekeeping officers to act as a part of the care team. The police officers’ role in de-escalation of patient agitation made reinforcement of treatment seamless for medical staff and, ultimately, a better outcome of care for the patient. By seeing each of the individuals involved in the patient’s case as potential teammates, patient care can be improved [10,11].

## Conclusions

This case study discusses the care and management of a patient with concurrent rhabdomyolysis and schizoaffective disorder. Through conservative management, the patient was treated for acute rhabdomyolysis and began treatment of his schizoaffective disorder. While often not considered a part of the care team, the police officers and their actions were key for the patient's acute recovery. This key point alone demonstrates that to ensure that the patient receives the best care, often, care teams need to be built with the patient in mind and what will ensure their individualized recovery, first and foremost. Because of the patient's psychiatric state, the involvement of the police officers was the best course of action to ensure that he was able to receive all the necessary treatment.
